# Leading edge analysis of transcriptomic changes during pseudorabies virus infection

**DOI:** 10.1016/j.gdata.2016.09.014

**Published:** 2016-09-30

**Authors:** Damarius S. Fleming, Laura C. Miller

**Affiliations:** Virus and Prion Research Unit, National Animal Disease Center, USDA, Agricultural Research Service, Ames, IA, USA

**Keywords:** Gene expression, Pseudorabies virus, Swine, Leading edge analysis, Specifications, Organism/cell line/tissue, *Sus scrofa* domesticus/tracheobronchial lymph nodes (TBLN), Sex, Male, Sequencer or array type, Illumina HiSeq 2000, Data format, Raw Digital Gene Expression Tag Profiling sequences, Experimental factors, infected with feral isolate FS268 of Pseudorabies virus vs. uninfected at 1, 3, 6, and 14 dpi, Experimental features, Very brief experimental description, Consent, N/A, Sample source location, N/A

## Abstract

Eight RNA samples taken from the tracheobronchial lymph nodes (TBLN) of pigs that were either infected or non-infected with a feral isolate of porcine pseudorabies virus (PRV) were used to investigate changes in gene expression related to the pathogen. The RNA was processed into fastq files for each library prior to being analyzed using Illumina Digital Gene Expression Tag Profiling sequences (DGETP) which were used as the downstream measure of differential expression. Analyzed tags consisted of 21 base pair sequences taken from time points 1, 3, 6, and 14 days' post infection (dpi) that generated 1,927,547 unique tag sequences. Tag sequences were analyzed for differential transcript expression and gene set enrichment analysis (GSEA) to uncover transcriptomic changes related to PRV pathology progression. In conjunction with the DGETP and GSEA, the study also incorporated use of leading edge analysis to help link the TBLN transcriptome data to clinical progression of PRV at each of the sampled time points. The purpose of this manuscript is to provide useful background on applying the leading edge analysis to GSEA and expression data to help identify genes considered to be of high biological interest. The data in the form of fastq files has been uploaded to the NCBI Gene Expression Omnibus (GEO) (GSE74473) database.

## 1 Direct link to deposited data

Raw sequence data for this study is available at: http://www.ncbi.nlm.nih.gov/geo/query/acc.cgi?acc=GSE74473.

## 2 Experimental design, materials and methods

### 2.1 Experimental design

The experimental design used in the original study is described in full in Miller et al., 2015 [1] and consisted of RNA isolation from porcine tracheobronchial lymph node (TBLN) tissue from infected and non-infected pigs. Pathogen-free pigs, between 4 and 5 weeks of age (*N* = 40) were split into two equal (*n* = 20) treatment groups and received intranasal inoculations of either a sham inoculum or a 1 × 10^6^ cell culture infectious dose (CCID_50_) of Pseudorabies virus Florida strain isolate 268 (FS 268). Tissue collections of porcine TBLNs were performed on necropsy days 1, 3, 6, and 14 dpi on five pigs from each of the treatment groups and stored at − 80 °C in RNA later. Total RNA extractions were conducted using 1 g of TBLN per pig in the MagMAX™-96 for Microarrays Total RNA Isolation Kit (Applied Biosystems). RNA sample quality was verified by both Bio-analyzer 2100 and RNA 6000 Nano-chip (Agilent Technologies) and had an RNA integrity number (RIN) of 7.8 on average for the extracted samples and a 28S/18S ratio of 1.9.

### 2.2 Digital gene expression profiling and sequencing

Tag preparation and library construction was performed using the Illumina DGETP *Dpn*II sample prep kit. One milligram aliquots of total RNA were used in accordance to the sample kit protocols to first isolate the polyadenylated RNA leading to cDNA synthetization, *Dpn*II digestion, and GEX DpnII adaptor ligation to the 5′ end of the cDNA fragments. Restriction sites 17 bp downstream from the adaptor were then cleaved with *Mme*I and a second adaptor ligated to the tag site. A 15-cycle PCR using complimentary primers was used for amplification of the cDNA fragments. After elution from the gel, the DNA was precipitated using 10 μL of 3 M sodium acetate (pH 5.2) and 325 μL of ethanol (− 20 °C), centrifuged for 20 min (14,000 RPM) and washed with 70% ethanol prior to resuspension in 10 μL of 10 mM Tris–HCl (pH 8.5). Quality was accessed using a Nanodrop 1000 spectrophotometer. Samples were then sequenced using the Solexa/Illumina Genome Analyzer II to generate a total of 8 raw fastq sequence files available in the public repository Gene Expression Omnibus (GEO) (GSE74473).

## 3 Transcriptome analysis

Analysis of transcriptional data based on identification and quantification of transcript tags for each transcriptional unit (TU) was carried out in a four step process. Step 1 utilized a custom Perl script to identify and filter tagged transcripts into a 3 column list generated from the first 20 bases of the tag sequence, the raw tag count, and normalized tag counts. Normalized tag counts were based upon total number of DEGTP tags for a TU divided by the total number of TU counts for a tissue then normalized as tags per million (TPM). Step 2 computed the transcript abundances for infected and non-infected samples quantification using the Audic-Claverie algorithm [2] that allows for calculation of the nominal *p*-values between infected and control groups based upon Bayesian averaging to infer the Poisson distribution of the tags. The Audic-Claverie algorithm was used to compare the 2 groups as control vs. infected for each dpi. The values for steps 1 and 2 were used as inputs for MatLab to calculate transcript abundance. In MatLab, an FDR of *≤* *0.01* was applied and tagged transcripts showing a two-fold or greater increase were considered to be differentially expressed. The tags displaying differential expression were then grouped using K-means clustering. Step 3 used the differential expression and K-means clustering information to perform a ranked gene set enrichment analysis (GSEA). A leading edge analysis was performed during this step to elucidate key genes related to both the transcriptomic changes and clinical signs related to PRV pathogenesis. Step 4 involved the visualization of the data as hive plots. Hive plot creation was used as a graphical representation of the transcriptomic changes witnessed in the PRV infected pigs and to allow for a means to accomplish direct infected/non-infected comparisons. The plots are organized to allow for readers to observe what genes are involved in which pathways and networks dependent on the day post infection. The hive plots are created from the intersection of the results for each dpi based upon the differentially expressed gene transcripts, the gene position, and GSEA results.

### 3.1 Leading edge analysis

**Fig. 1:**
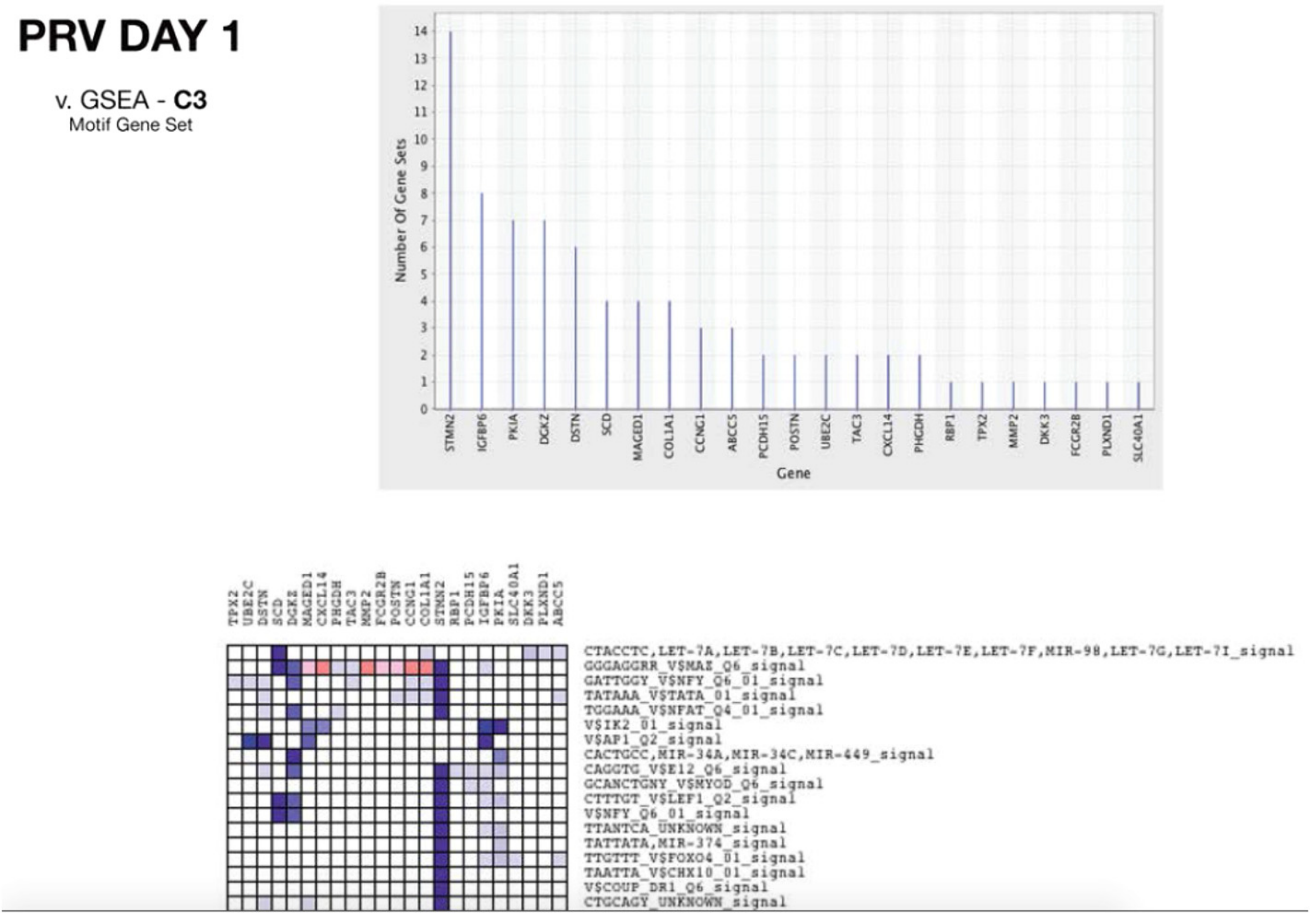
Example from 1 dpi showing the graphs for the “Heat map” and “Gene in Subsets” output from the leading edge analysis module. Input is based on the motif gene set from the GSEA. These leading edge analysis graphs were used in the current study to identify candidate genes possibly connecting clinical PRV signs to transcriptomic changes.

In order to determine which genes have the highest impact on the biological process under study, a portion of the GSEA was dedicated to performing leading edge analysis of the differentially expressed genes. The leading edge analysis allows for the GSEA to determine which subsets (referred to as the leading edge subset) of genes contributed the most to the enrichment signal of a given gene set's leading edge or core enrichment [3]. The leading edge analysis is determined from the enrichment score (ES), which is defined as the maximum deviation from zero [1], [3]. The analysis is accomplished by setting the GSEA software parameters to define subsets of the core genes that drive the enrichment score of the GSEA clusters. Step 1 of initiating the leading edge analysis is to select the gene sets from the GSEA results that are to be compared. This can be done by ranking the gene sets by an FDR cut-off. For our study this was represented by the enriched gene sets for each of the dpi (1, 3, 6, 14). In step 2, the GSEA software will output four graphs representing the overlapping subsets of the chosen gene sets (i.e. subsets for each dpi) from step 1. The four graphs that are generated are: (a) Heat map which shows the clusters and expression values for the leading edge subsets color-coded to represent ranges from lowest to high (Fig. 1), (b) Set-to-set graph that displays the overlap between the subsets in which the number of genes shared between subsets is displayed as color intensity. The intensity of the color is directly correlated to amount of overlap, (c) Genes in subset list (Fig. 1) which is a simple graph of how many subsets in which a particular gene belongs. This can inform the researcher of key candidate genes whose functions may be of biological interest. The last graph (d) is a histogram showing the Jacquard, which gives the number of subset occurrences binned by frequency. This will give information on how many subset pairs share overlap. Step 3 gives the researcher the ability to initiate the “Build HTML Report” which will give all of the details for interpreting the leading edge analysis [4]. The genes that comprise a leading edge subset have a high correlation between their expression level and the phenotype in question and tend to be at the extremes of the distribution, rather than randomly distributed. This subset is essentially the genes within each cluster responsible for the enrichment score for that cluster. This is based on several statistical values referred to as the Tag, List, and Signal. The tag is the number of genes of the leading edge subset that actively contribute to the enrichment score, the list gives the position or rank of the genes, and the signal is the strength/intensity of the genes. A key use of this GSEA module is to examine the overlap in enriched genes between groups. The comparison of the overlap can be extended over time points to better understand what genes tend to be involved at the core of the transcriptomic response during infection. In our study, comparing the output from this analysis at each dpi allowed for the ability to rank subsets by expression and enrichment level and compare this to the daily progression of PRV clinical symptom pathology. The combinatorial effect of the GSEA, DEG, and leading edge analysis output gave our study means for observing what genes and which genetic (transcriptomic) changes are reflected by the disease phenotype.

## 4 Conclusion

A major goal of this study was to profile the biological and molecular networks involved in the pathological response caused by PRV infected TBLN. The analysis pipeline that was used also gave the study the ability to relate biological networks to the clinical progression of PRV observed in the animals at each dpi (1,3,6,14). Taken along with the results from the leading edge analysis, the study was able to provide data on which genes were being differentially expressed but also allowed for the recognition of the number of gene sets and key genes within those sets, whose expression varied significantly as PRV pathology progressed from 1 dpi to 14 dpi. Leading edge analysis was used to determine the genes that overlapped between treatment groups and dpi's that contribute the greatest to the transcriptomic response to PRV. These leading edge or core genes are considered to be of high biological interest due to appearing at higher frequencies among the subsets between groups.
